# On the identification of sensory information from mixed nerves by using single-channel cuff electrodes

**DOI:** 10.1186/1743-0003-7-17

**Published:** 2010-04-27

**Authors:** Stanisa Raspopovic, Jacopo Carpaneto, Esther Udina, Xavier Navarro, Silvestro Micera

**Affiliations:** 1 ARTS Lab, Scuola Superiore Sant'Anna, Piazza Martiri della Liberta' 33, Pisa, Italy; 2 Institute of Neurosciences and Dept. Cell Biology, Physiology and Immunology, Universitat Autònoma de Barcelona (UAB), E-08193 Bellaterra, Barcelona, Spain; 3 Centro de Investigación Biomédica en Red sobre Enfermedades Neurodegenerativas (CIBERNED), E-08193 Bellaterra, Barcelona, Spain; 4 Institute for Automation, Swiss Federal Institute of Technology, ETL K 10 Physikstrasse 3, Zurich, Switzerland

## Abstract

**Background:**

Several groups have shown that the performance of motor neuroprostheses can be significantly improved by detecting specific sensory events related to the ongoing motor task (e.g., the slippage of an object during grasping). Algorithms have been developed to achieve this goal by processing electroneurographic (ENG) afferent signals recorded by using single-channel cuff electrodes. However, no efforts have been made so far to understand the number and type of detectable sensory events that can be differentiated from whole nerve recordings using this approach.

**Methods:**

To this aim, ENG afferent signals, evoked by different sensory stimuli were recorded using single-channel cuff electrodes placed around the sciatic nerve of anesthetized rats. The ENG signals were digitally processed and several features were extracted and used as inputs for the classification. The work was performed on integral datasets, without eliminating any noisy parts, in order to be as close as possible to real application.

**Results:**

The results obtained showed that single-channel cuff electrodes are able to provide information on two to three different afferent (proprioceptive, mechanical and nociceptive) stimuli, with reasonably good discrimination ability. The classification performances are affected by the SNR of the signal, which in turn is related to the diameter of the fibers encoding a particular type of neurophysiological stimulus.

**Conclusions:**

Our findings indicate that signals of acceptable SNR and corresponding to different physiological modalities (e.g. mediated by different types of nerve fibers) may be distinguished.

## Background

In the recent past, several groups have worked on the development of neuroprostheses to restore sensory-motor functions lost in patients affected by spinal cord injury or stroke [1-3]. A number of these neuroprostheses use functional electrical stimulation (FES) to elicit the contraction of different muscles that are no longer controlled by the central nervous system in order to obtain functional movements. Although interesting results have been achieved in the activation of lower extremity motion and control of hand movements [4-7], various problems still exist since, in most cases, FES is delivered in open loop and does not take into account factors such as the dynamic time-variant properties of the musculo-skeletal system. This issue can be addressed by developing closed-loop control algorithms based on the extraction of sensory information, and its use for correcting deviations caused by unexpected changes and non-linearities. Feedback information can be gathered by using implantable [8,9] or external [10,11] artificial sensors or by processing electroneurographic (ENG) signals recorded by means of implanted interfaces with the peripheral nerves of the subject [12]. In the latter case, the choice of the electrode will make a difference on the type of processing available based on the selectivity of the electrode and its placement. For example, by using cuff electrodes only the superposition of action potentials belonging to many different axons activated in the same nerve can be identified. Thus, the contribution of single axons could be difficultly extracted because of the low signal to noise ratio (SNR) and of the possible overlapping between signal frequency ranges (few hundred Hz to a few kHz) and noise [12].

In most cases the use of recorded neural activity has been limited to sensory event onset detection for the closed-loop control of FES systems [13-15] and for the control of hand prostheses [16,17]. These limits can be partly overcome by using multi-site cuff electrodes [18], but it would still be important to enable strategies for discriminating sensory information that can be extracted from ENG signals recorded in a whole nerve using simple cuff electrodes.

Cuff electrodes have been used for more than thirty years [19] to stimulate peripheral nerves and also to record electroneurographic (ENG) signals. Interestingly, Haugland and coworkers [13-16] demonstrated that sensory events, such as skin contacts or slip information, could be recognized with respect to the background rest-noise from cuff recorded neural signals in cats as well as in humans. However, the main goal of these studies was to identify the onset (and offset) of a specific neural activity, with the aim of triggering stimulation. The aim of our work was to investigate the ability to discriminate different types of sensory stimuli from the nerve signals recorded by using a cuff electrode [20], and to propose an optimal signal processing scheme. In particular, artificial intelligence classifiers were used to discriminate different features extracted from afferent signals, evoked by different types of sensory stimuli and recorded with a cuff electrode placed around the rat sciatic nerve. Our hypothesis is that at least two stimuli can be discriminated with good performance, and that classification performance depends on the quality of neural signals recorded, which in turn is related to the diameter of the fibers encoding a particular type of neurophysiological stimulus.

For such purpose, particular attention must be devoted to the selection of the features to be extracted. Whereas several previous works have described the features to be extracted from electromyographic (EMG) signals and from intraneurally recorded ENG signals (e.g. using longitudinal intrafascicular electrodes and multielectrode arrays), only a few studies have addressed this issue for extraneurally recorded ENG. In fact, ENG signals obtained by means of single-channel cuffs can be considered roughly in between cumulative EMG signals and highly selective intraneural ENG signals.

In this paper, the features proposed in previous works using single-channel cuff electrodes [21-24], as well as those proposed in studies on EMG [25-27] signals were analyzed in order to find the most informative feature combination to feed into the classifiers. Finally, in order to explore eventual presence of bursting nerve activity (superposed to the background signal and not detectable by visual perception) a wavelet denoising method, which allowed the classification of spikes from neural signals recorded using invasive intraneural electrodes [28,29], was also tested.

## Materials and methods

### A. Experimental setup

Tripolar polyimide cuff electrodes (with three parallel ring Pt electrodes), with an inner diameter of 1.2 mm and a length of 12 mm were used. The fabrication process and in vivo use have been described in detail previously [20]. The polyimide-based microstructure consists of a flat rectangular piece (12 × 6.75 mm) - containing the electrode contacts and rolled into a cylinder spiral shape - and an interconnect ribbon (2 mm wide, 26 mm long) with integrated contacts attached to a ceramic connector.

**Figure 1:**
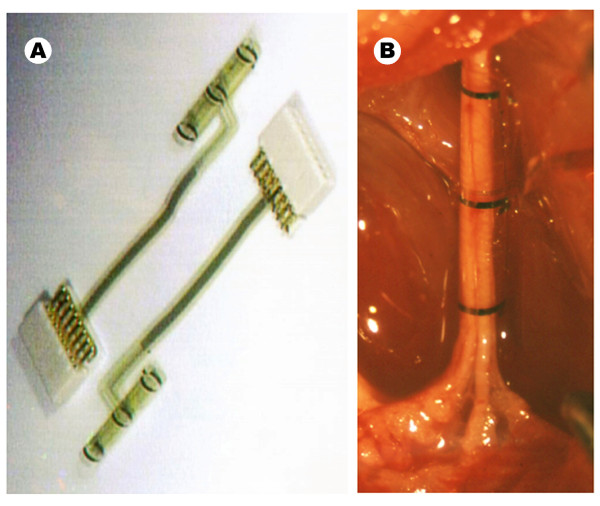
**Polyimide tripolar cuff electrode used in the study**. Cuff electrode and connector (A), and its implantation around the sciatic nerve of a rat before performing the experimental study (B).

Experiments were performed in five Sprague-Dawley rats. Under general anesthesia with ketamine/xylazine (90/10 mg/kg i.p.), and with the aid of a dissecting microscope and microsurgery tools, the sciatic nerve was exposed at mid-thigh and carefully freed from surrounding tissues. The cuff was opened and placed around the sciatic nerve avoiding compression and stretch. After release, the spiral cuff was closed covering the whole nerve perimeter (Figure 1).

Since the animals were under anesthesia during the study, the problems related to the presence of movements previously experienced [13,14] were mainly avoided. Therefore, this represents an "optimal" condition for detecting solely afferent activities, with minimal or absent muscle artifacts.

All experiments were performed inside a Faraday cage, in order to minimize the amount of electromagnetic noise interfering with the recordings. The experimental procedures adhered to the recommendations of the European Union and the NIH Guide for Care and Use of Laboratory Animals, and were approved by the Ethical Committee of the Universitat Autònoma de Barcelona, where the animal work was performed.

### B. Stimuli application and signal recording

Different sensory stimuli were applied to discrete areas of the hindpaw and the evoked neural activity was continuously recorded. Three different types of stimuli were sequentially applied, ten times each, to each animal: (1) mechanical stimulus ("VF") of regulated intensity by touching the plantar skin with a von Frey filament (Stoelting Co, Illinois) (2) proprioceptive stimulus ("Proprio") provoked by means of complete passive flexion of the toes, and (3) nociceptive stimulus ("Nocio") provoked by pinching the toes. These three types of stimuli were selected because they elicit impulses conducted by three different functional classes of afferent nerve fibers (A*β *tactile mechanoreceptive, A*α *proprioceptive, and A*δ*/C nociceptive, respectively).

Efforts were made to standardize the intensity of stimuli across trials: the same Von Frey filament was used in all the tests, thus providing the same contact pressure; passive flexion was produced by bending the toes from the horizontal plane to about maximal flexion by means of small wood sticks that were glued to the dorsum of the nails, to avoid tactile stimulation; pinching the toe was made using the same fine forceps (Dumont #5), aiming to elicit pinching pain, with minimal touch.

Onset and duration of stimuli were identified by experimenter's bottom pressure in synchrony with start and end of stimulus application, while VF touch stimulation was also recorded by means of a pressure sensor located under the animal hindpaw, confirming good timing given by means of bottom pressure. The duration of different stimulus applications were not statistically different, and had small standard deviation (touch stimulus (mean ± standard deviation): 0.96 ± 0.11 sec; proprioceptive: 1.17 ± 0.18 sec; nociceptive: 0.97 ± 0.25 sec).

**Figure 2:**
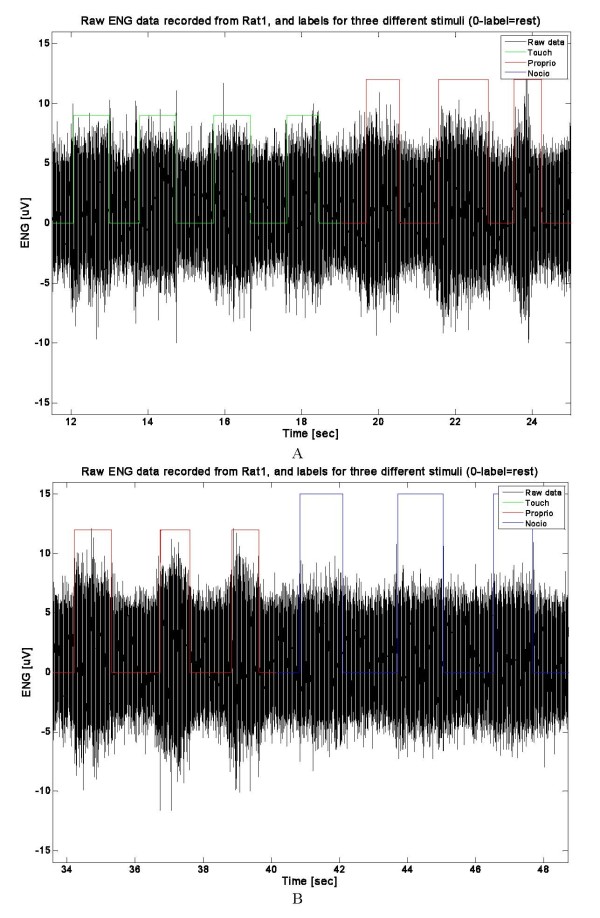
**Examples of raw ENG recordings**. In black is presented raw voltage; green labeled steps represents application of Touch stimulus; red labeled steps represent Proprioceptive stimulus application; in both cases the label with value 0 represents absence of stimulus (A). In black is presented raw voltage; red labeled steps represents application of Proprioceptive stimulus; blue labeled steps represent Nociceptive stimulus application; in both cases the label with value 0 represents absence of stimulus (B).

Neural signals (Figure 2) were differentially amplified (at 10,000X; Isolated Microamplifier, FHC Inc.), analogically filtered (band pass filter with cutoff frequencies of 10 Hz and 5 kHz), digitized at 20 kHz (PowerLab) and fed into a PC running Chart v5.5 (AD Instruments). Datasets consisted of ten applications of every type of stimuli during the experiment. Noisy parts of the recordings - corresponding to stochastic nerve and muscle discharges - were not eliminated since they would be present also in any real prosthetic applications. In this way, the experiments should be able to indicate the real limits of this approach.

### C. Signal processing steps

**Figure 3:**
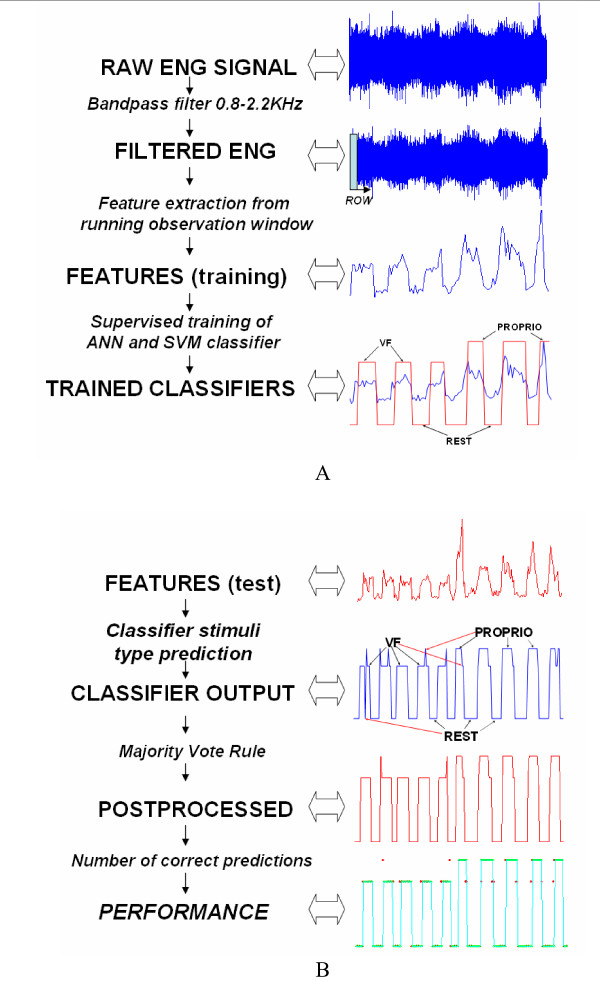
**Block diagram of the proposed classification system for ENG signals**. Training is performed on the first and testing on the second half of the data. (A) Training procedure consisting of: Filtering (in order to eliminate the EMG low band, and amplifiers high band noise); Feature extraction from running observation window (ROW); Training of the classifier with stimuli type knowledge (VF, Proprioceptive, Rest as labeled); recorded during the experimentation. (B) Test procedure: filtering and feature extraction are first steps (both the same as during the training); then the classifiers (trained in A) answer is post-processed by means of majority vote rule. Evaluation is carried out by report between correctly classified instances and all samples in each test set.

Figure 3 shows a block diagram of the proposed classification scheme. Panel A describes the steps implemented during the training phase. pre-processing, feature extraction, training of the classifiers using a supervised approach. Panel B illustrates the steps performed during the test phase: pre-processing, feature extraction (both the same as during the training), identification of the stimulus using the trained classifier, and a majority voting technique. The different steps are described in detail in this section.

#### Signal Pre-processing

Initially, a preliminary spectral analysis was performed in order to impose correct filtering. Consistent with previous results [13], a neural signal peak between 1.0 and 2.0 kHz was observed for all the stimuli-evoked responses analyzed. In a previous study [22], the nerve cuff signals were found to be independently distributed Gaussian signals with zero mean and modulated in variance. Consequently, the ENG signals recorded during the different experimental conditions were digitally filtered using a FIR bandpass filter with 0.8 KHz and 2.2 KHz cutoff frequencies in order to reduce the presence of undesired signals (e.g. low frequency EMG signals and high frequency amplifier noise). In fact, about 95% of the power spectrum of the EMG is accounted for by a band up to 400 Hz - although there are some harmonics up to 800 Hz [25] - while amplifier noise makes an important contribution only at higher frequencies [21].

#### Length of running observation window and overlap

In this kind of signal processing paradigm, one of the parameters to choose is the optimal length of the running observation window (ROW), and possible overlap. In EMG studies, the plateau in classification performance for observation windows starts from 100 ms [30,31]. Since there are no indications in the literature either for optimal window length with ENG signals or for overlap (allowing a greater amount of samples for post-processing rule [30,31]), the identification of these parameters was analyzed first. Therefore, different observation window lengths were studied [25, 50, 75, 100, 125, 150, 200, and 300 ms], and for the best performing lengths, different overlaps [1/4, 1/2, 3/4] were tested.

Feature extraction. Several features were extracted from the ENG signals (see Table 1 for mathematical definitions and references), in an attempt to enhance the ENG signals conveying different sensory information with respect to the resting-state ENG.

**Table 1:** Mathematical definitions of the features used in this study

Feature Name	Definition	Description and references
**Mean absolute value (MAV)**		[25]
**Variance (VAR)**		[25]
**Wave length (WL)**		[25]
**Wavelet denoise (WDEN)**	*θ *= *σ *(0.3936 + 0.1829log_2_(*N*)), *σ*-standard deviation of noise	Symmlet 7 mother wavelet, and hard threshold (*θ*). [28,29]. Finally the feature is MAV of denoised signal.
**Energy based on discrete Fourier transformation (DFT)**	, where X [k] is DFT of x [n]	[25]
**Autoregressive coefficients (AR)**		The forward-backward approach. The sum of a least squares criterion for a forward model and the analogous criterion for a time-reversed model is minimized [27].
**Cepstral coefficients (CEPS)**	c_1 _= -*a*_1_	The cepstrum coefficients (c_i_), are calculated from AR coefficients (AR model with order P), as proposed in Kang's work [26].
**Autocorrelation-based, second order processing (HOS2)**	, H_0_-noise only (null hypotesis), H_1_-presence of signal	Toepliz matrix creation, based on estimate of autocorrelation; singular value decomposition; difference among maximum and minimum eigenvalue (*σ*) [21].
**Cumulant-based third order processing (HOS3)**	, H_0_-noise only, H_1_-presence of signal	Toepliz matrix creation, based on estimate of third order cumulant of a data frame; singular value decomposition; the largest eigenvalue (*λ*) [21].
**Autocorrelation minimum value (ACORR)**	, L-length of ROW	First negative peak value of r(*τ*) [23,24].

References are also provided for further explanation. N is the number of samples in ROW and xi is the single sample.

First, standard, time domain features used to process EMG signals were estimated from the ROW: mean absolute value (MAV), variance unbiased estimator (VAR), and wave length (WL) [25].

Then, the features proposed in the few previous studies on single-channel cuff ENG processing were tested. In [21] a higher order statistics approach was proposed, which is able to separate the space of the noise with respect to the space of the signal of interest. Briefly, this means: a) constructing the Toeplitz matrix based on second order estimation (autocorrelation) (HOS2) or third order statistics (HOS3); b) transforming it into the eigenvalues matrix, by means of singular values decomposition, and c) taking the values higher than an empirical threshold.

On another hand, [23,24] proposed to use the autocorrelation function to distinguish different activities by analyzing whole nerve signals recorded with cuff electrodes, based on the differences in fiber conduction velocity. Five possible factors may be extracted from this feature (ACORR): zero-cross time, time of minimum, minimum value, time of maximum, and maximum value. We tested these five parameters and found that the first minimum value showed the greatest difference between noise and elicited ENG activity.

Energy based on Discrete Fourier Transformation (DFT) of the signal was used to understand whether our ENG signals are more separable in the frequency domain [25].

Features based on time-series analysis have already shown to be useful in EMG signal processing, hence cepstral (CEPS) [26], and autoregressive (AR) [27] coefficients were included in the present study.

Finally, a wavelet-denoise with hard-thresholding and Symmlet 7 mother wavelet (WDEN) was implemented [28,29], in order to extract the bursting activity, possibly superimposed to compound signals and not identifiable visually. All these features were extracted from the ROW, and were used as inputs to the classification systems.

#### Classification algorithms

The above features were normalized with respect to the corresponding maximal values, and were used as inputs to two non-linear classifiers applied in this study:

1. An artificial neural network (ANN) [32]: a feed-forward neural classifier, trained by back-propagation rule, comprising two hidden layers with 10 neurons was used. Since there is no standard way to define the appropriate topology of a neural network nor the number of neurons, the parameters were determined by means of iterative search. The numbers of hidden layers (from 1 to 3) and neurons (from 1 to 11), and the optimal topology and number were found with respect to the peak of classification accuracy (this is not shown in the manuscript for the sake of brevity). The optimal configuration used had two hidden layers with 10 neurons each. The input layer was composed of neurons corresponding to the number of features used during simulations (from one to four), while in the output layer there were four neurons, related to the possible states-classes of the problem (rest, mechanical stimulus, nociceptive stimulus and proprioceptive stimulus).

2. Support vector machine (SVM) classifier [33] maps input data into the feature space where they may become linearly separable. Due to its superiority in terms of good generalization derived from minimizing structure risk, SVM has been applied successfully in bio-information and pattern recognition [29,31,34]. The SVM network was investigated using Gaussian Radial Basis function (RBF) kernel, which yielded the best results during preliminary investigations. A grid-search was employed as a method of model selection to adjust SVM parameters, as proposed in [31,34]. In this method, the performance of a SVM was examined based on a wide range of parameters; then the fitter grid-search was implemented close to parameters yielding best results. A four fold random cross validation scheme was used to evaluate the parameters. Recently, this kind of classifier was used in EMG signals classification [31] (further details on SVM theory may be found here).

The training process for the ANN was not repeatable since it was initiated from random initial weights, and sought local minimum errors rather than global ones. Instead, for the SVM it was repeatable and fast. The SVM can settle to a global minimum error after training.

#### Majority vote post-processing

As the last step, majority vote (MV) post-processing [30,31] was applied. The MV is a post-processing that eliminates transient jumps, and produces a smooth output. It counts the estimated classes in the 2 *k *+ 1 estimations about a considered estimation (*k*-estimations before and *k*-estimations after), and outputs the value that occurs most as a corresponding estimation. Thus, the value of the final output is the class with the greatest number of occurrences in this point window of the decision stream. The number of samples (k), that can be used in the majority vote was determined by the processing time, overlap used and acceptable delay. Processing time is the time needed to make a decision after the observation window (e.g. filtering, feature calculation and pattern classification) and depends on the type of microcontroller or digital signal processor used in the real time prosthetics system. This time should be within a few milliseconds. Overlap is the time of the overlap between two ROWs. Acceptable delay (i.e. not perceivable by the user) would roughly be between 175 and 300 ms [30,31,35]. Since MV uses the next *k*-estimation to produce the current output and avoid any failure in real-time control, it is possible to determine the maximum number of decisions to use within the MV rule. Hence, real-time constraints impose (considering 0 ms processing time):(1)

where ROW is the length of the running observation window (ms), and Olap is the overlap between two consecutive running observation windows.

### D. Evaluation

In order to validate the results of the classification models, the first half of the signals was used to train the parameters of the classifiers; their performance was then assessed on the second half of the data. The performance of the classifiers was measured by comparing the number of correctly classified instances with the total number of instances within the test set.

Statistical analyses were applied to interpret the experimental results. The purpose of statistical analysis was to find statistically meaningful differences between observations with a certain significance. Due to the relatively low rate of observations and their unknown distribution, nonparametric approaches were applied. Kruskal-Wallis is an extension of the Wilcoxon rank-sum for data with more than two groups, and is suited for this type of analysis. The critical p-value, which determines whether a result is statistically significant, was 0.05.

## Results

For all the datasets, the SNR was calculated as the ratio between the mean MAV amplitude of the ENG signals recorded whilst stimulating the animal hindpaw and the mean MAV amplitude recorded during absence of any stimulation (resting period), [21]:(2)

**Figure 4:**
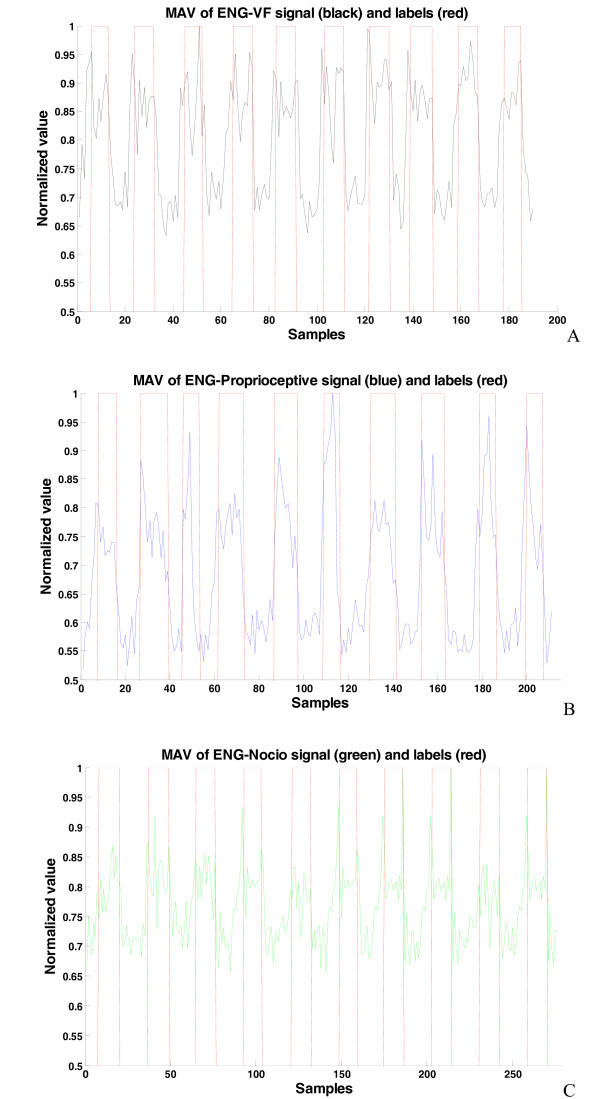
**MAV of three types of stimuli, used for SNR calculation for Rat1 dataset**. A) VF stimuli, B) Proprioceptive stimuli and C) Nociceptive stimuli. In red are presented the labels, corresponding to time of stimulus application, used for supervised training.

The results, for the Rat1 dataset, can be seen in Figure 4. The results shown in Table 2 indicate a relatively low SNR (only a few decibels), ranging from 1.2 to 3.8 dB. Proprioceptive stimulation provided the best SNR levels among the three stimuli, and tactile stimulus had better SNR compared to pain stimulation. These SNR values are proportional to the diameters of fibers conducting the corresponding stimuli.

**Table 2:** Calculated signal to noise ratio (SNR) of ENG signals corresponding to the different stimuli

Animal	SNR (VF) [dB]	SNR (Proprioceptive) [dB]	SNR (Nociceptive) [dB]
**Rat 1**	2.4380	3.8230	1.4292
**Rat 2**	2.1937	3.6601	1.8584
**Rat 3**	1.9592	2.2802	1.6879
**Rat 4**	2.0592	3.0752	2.0530
**Rat 5**	1.5140	2.2923	1.1692
**Mean ± Standard Deviation**	2.0328 ± 0.3411	3.0262 ± 0.7305	1.6395 ± 0.3488

Stimuli (Von Frey (VF), Proprioceptive, Nociceptive) applied in the five rats used in the study.

For the complete datasets of the five rats, signal processing was performed with the aim of identifying the median and upper limits of afferent stimuli discriminable, and the optimal values for the data-processing scheme (e.g. ROW, overlap, features and classifier choice). The pattern classification ability to discriminate the different stimuli was tested starting from only one stimulus w.r.t. rest state, and progressively increasing the number of stimuli to be identified on groups of two and three, until finding an acceptable percentage of classification. The influence of different parts of the proposed signal processing scheme, and recommendations on optimal choices are given below.

### Running observation window (ROW), overlap length and majority vote rule

**Figure 5:**
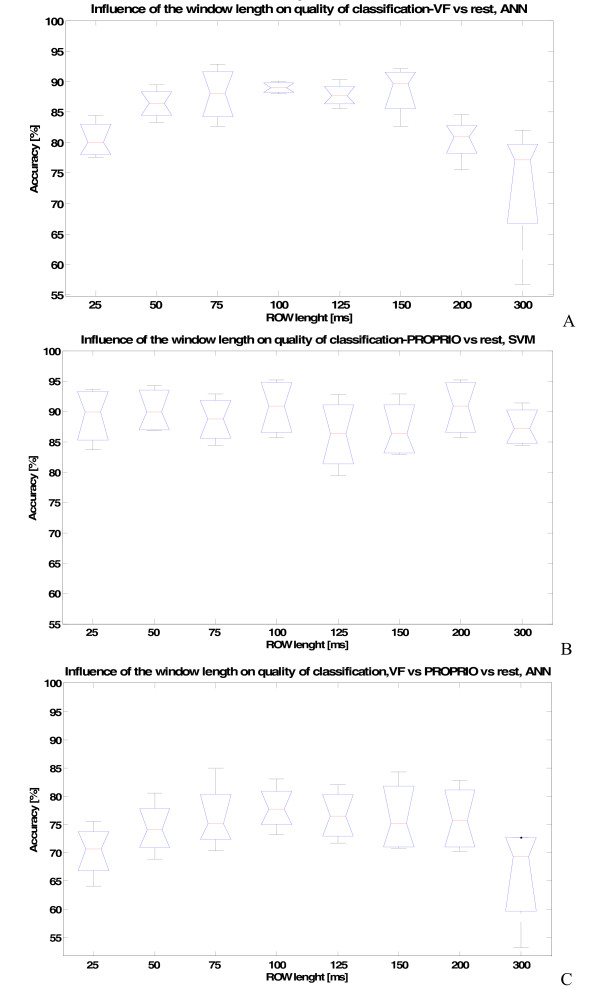
**The influence of Running Observation Window length (ROW) on the quality of classification**. The case of: A) VF versus rest stimuli, (MULTI1 = (MAV + WL) features set), ANN classifier, B) Proprioceptive versus rest stimuli (MAV feature), SVM classifier, and C) VF versus Proprioceptive versus rest, (MULTI2 = (MAV + VAR + WL) feature set), ANN classifier. The peak of performance can be observed for 100 ms, indicating that this is the optimal value.

For all stimuli and stimuli combinations, feature combinations and different window lengths were tested, in order to define the optimal length for this kind of signals (Figure 5).

The trend for different features and for different stimuli was found to be similar: as expected, the information contained in features is not stable enough (too biased) for short windows (e.g. 25, 50 ms), while, in contrast to EMG-studies [30,31], a decline in performance was observed for excessively long windows (e.g. 300 ms). Peak performance was in the 100 ms window in most cases (Figure 5). While for almost all combinations of stimuli, the median of performances had a 100 ms length peak, for VF versus rest stimulus the 100 ms ROW was also significantly different w.r.t. the 25 ms and 300 ms ROW (p < 0.05). These results indicated that the 100 ms ROW was optimal for the next processing steps.

**Figure 6:**
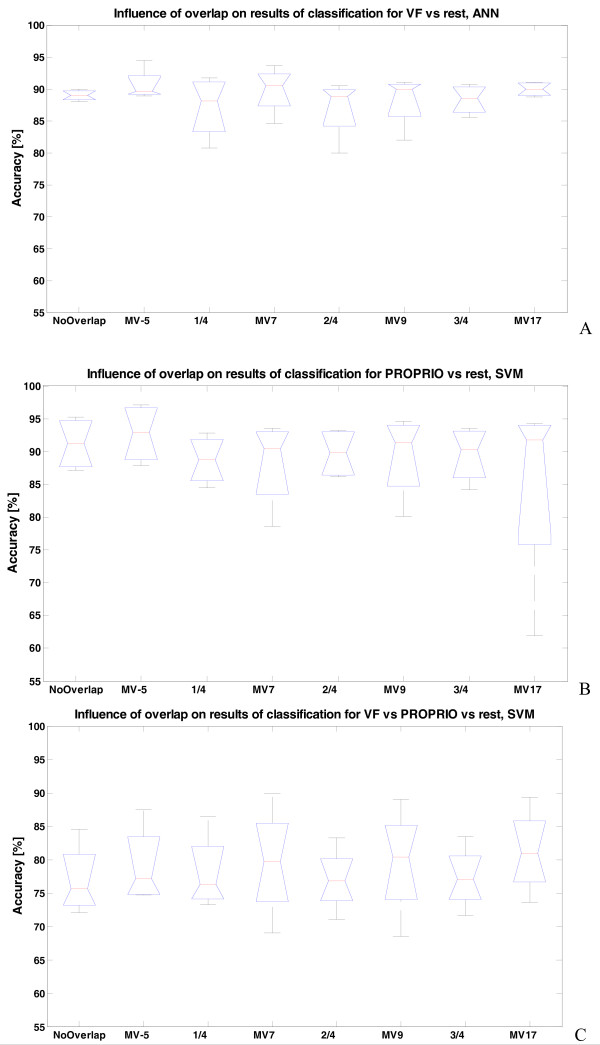
**The influence of overlap between ROW and general improvement of the performance when Majority Vote (MV) rule is used**. The case of: A) VF versus rest stimuli (MULTI2 feature), ANN classifier B) Proprioceptive versus rest stimuli (MULTI2 feature set), SVM classifier and C) VF versus Proprioceptive versus rest (MULTI2 features), SVM classifier. For 100 ms ROW, considering 0 ms processing time, the maximal permitted values of points to consider in majority vote (MV) are 5, 7, 9 and 17 for respectively: no overlap, 1/4 overlap, 1/2 overlap, and 3/4 overlap. Results are present in pairs showing median improvement when using MV: No overlap and No overlap with MV 5 applied (MV5); 1/4 ROW overlap (1/4) and 1/4 ROW overlap with MV 7 applied (MV7); 1/2 ROW overlap (1/2) and 1/2 ROW overlap with MV 9 applied (MV9); 3/4 ROW overlap (3/4) and 3/4 ROW overlap with MV 17 applied (MV17).

Therefore, with this ROW length, different overlaps were tested to find the appropriate decisions stream density, and majority vote with respect to quality of classification and permitted delay (Figure 6).

The results indicate that the majority vote post-processing rule enhances performance in every tested case. The most stable results were observed using disjoint windows, with majority vote based on five samples (MV5). Thus, this combination was used for studying the next, best features and classifier selection.

### Feature selection and classifier choice

**Figure 7:**
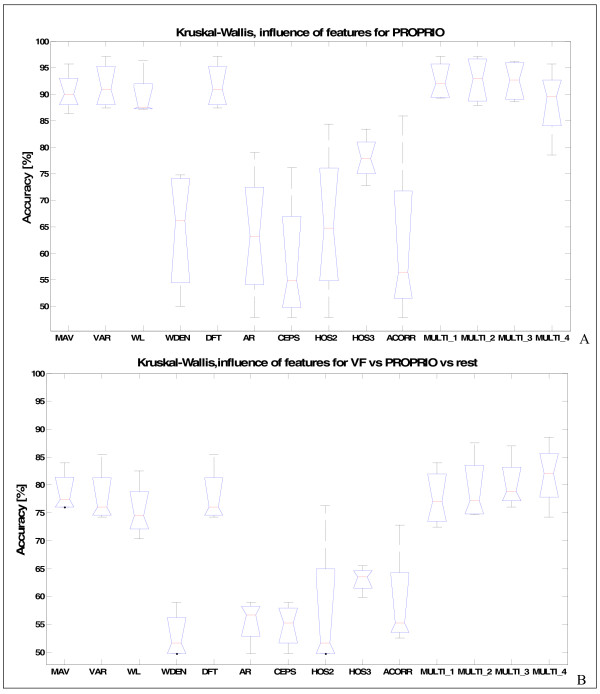
**The influence of different features on the performance of classification when using the 100 ms ROW, MV with 5 samples, and SVM classifier**. The case of: A) Proprioceptive versus rest; B) VF versus Proprioceptive versus rest. The "power-based" (MAV, VAR, WL, DFT) features and their combinations (MULTI1, MULTI2, MULTI3, MULTI4) are significantly better than the other tested (p < 0.05, Kruskal Wallis test). Among the best features there is not significant difference.

The statistical analysis was applied to the results of the classification for every single feature and for feature combinations tested, obtained using the optimal ROW (100 ms) and majority vote (MV5). The best performing features were combined so as to test whether the results could be improved: MULTI1 = MAV + WL; MULTI2 = MAV + VAR + WL and MULTI3 = MAV + VAR + WL + DFT. Moreover, we tried to combine good-performing features with other best-performing features (HOS3), in order to determine if they carried complementary information that would permit to obtain the best generalization: MULTI4 = MAV + WL + HOS3 (Figure 7).

The results indicate that "power-based" features (MAV, VAR, WL, DFT, and their combinations) performed significantly better w.r.t. others (p < 0.05). This trend was found for every stimuli and stimuli combinations. When, as a second step, the worst-performing features were eliminated, no statistical differences were observed between the good-performing features. The use of any "power-based" features, or any MULTI combination, gave similar results, but since they had slightly better median results, MULTI3 and MULTI4 are shown in the last step, aimed at finding the applicability of single-channel cuff electrodes for afferent discrimination.

Although the SVM classifier performed slightly better, no significant difference between the two classifiers was observed (p ≥ 0.05). The repeatability and speed of the training process, together with the better mean percentage of classification obtained indicate that SVM could be the optimal choice.

### Analysis of the median and maximal discrimination ability

After the identification of the most promising values for the different parameters of the classification algorithm, the ability of discriminating the different stimuli was investigated. In particular, the median and the maximum of the classification performance were extracted to analyze the robustness and the upper limit of the discrimination, respectively.

**Figure 8:**
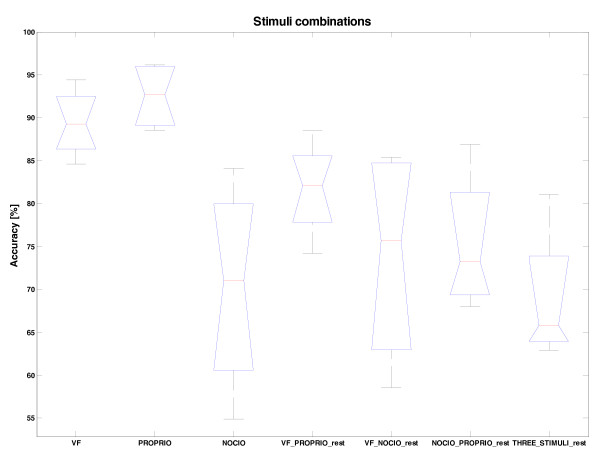
**Different stimuli and stimuli combination recognition achieved with the optimal proposed approach**. The values of different parameters used: ROW = 100 ms, MV5, SVM classifier, MULTI 3 set of features (in the case of VF vs proprioceptive, the MULTI4). VF and proprioceptive stimuli, as well their combination can be recognized from background rest-noise. The nociceptive stimuli conveyed by thin fibers, are difficult to recognize from background rest-nosy activity. In some cases (Rat1 dataset), corresponding to the maximal values, the three types of stimuli could be recognized.

The results (Figure 8) clearly indicate that single-channel cuffs could possibly be used for robust separation of proprioceptive, and touch-based, VF stimuli, from background rest-noise ENG, with above 90% median (and best performance of 97% and 95%, respectively, in the best dataset). Also their combination w.r.t. rest, could be discriminated reasonably well, with median performance above 80% (reaching 88%, in the best dataset). Nociceptive stimulus - conveyed by small pain fibers and featuring very small SNR - are not easy to recognize in a repetitive way.

As for the upper limit of discriminability, the maximal values in Figure 8., corresponding to the Rat1 dataset, should be observed. They indicate that, in case of good (repetitive) nociceptive stimulus (implant position and electrode coupling with the nerve were optimal), 80% could also possibly be achieved for three stimuli discrimination.

## Discussion

The technological improvement of motor neuroprostheses has led to an increased demand for fine control of devices. Providing sensory feedback of the controlled action is mandatory to improve the use of neuroprostheses in disabled subjects. However, due to the complexity of natural sensory systems, multiple artificial sensors should be needed to supply such information, needing for calibration and introducing bulkiness, with decrease of reliability [12,36]. The use of natural peripheral nerve afferents seems a better alternative, therefore, since they are available and functional in most patients affected by central nervous system injuries, who can benefit from the use of a neuroprosthesis [36]. The use of natural afferent neural activity requires a system capable of recording and differentiating the signals conveyed in a peripheral nerve in response to different types of stimuli. Due to their relatively low invasiveness, cuff electrodes seem well suited for implantation in the intact peripheral nerves of subjects [12]. Moreover, they can also be used to perform stimulation in FES systems, therefore the utility of their implant could possibly be double. However, neural activity recorded from peripheral nerves with a cuff electrode is usually of small amplitude and difficult to interpret. In this study, several processing methods were tested in order to optimize the classification (with acceptable processing delay) of ENG afferent signals recorded from the rat sciatic nerve using single-channel cuff electrodes.

Firstly, in the proposed signal processing paradigm, optimal factors for filtering, ROW length and majority vote use were found, indicating that 100 ms ROW with MV5 post-processing should be used. Then we addressed the problem of identifying optimal features in order to discriminate the sensory information from the ENG signals. Very few studies have been performed with this purpose. The autocorrelation method proposed by [23] can give good results when ENG has good SNR, but, as found in [24], it does not perform well in low SNR signals, generally encountered in this experimental study. Higher order statistics [21] are good for on/off detection [13-15] but not for the discrimination of different types of signals, while the wavelet denoise method, successfully used in intraneural recordings [28,29], was not able to find some specific, underlying data; these were the worst performing features. Other, basically "power-based" features performed similarly, without significant differences. Besides good performance in classification, they are easily implementable and do not imply excessive computer load.

The limiting factor for classification performance was found to be the SNR. It depends on many stochastic factors, such as positioning of the electrode, micro-damages and nerve orientation w.r.t. electrode, and also on the neurophysiological nature of signals (see Table 2).

An additional difficulty of the present study lied on the fact that inter-trial time (between two stimuli) was short compared to the duration of the stimulus application. This is a difficult situation for analysis, since it has been shown [37], that the amplitude of the afferent activity (and in consequence of SNR) increases with increasing inter-trial delay. Probably, with longer inter-trial time better results could be achieved, but we chose to study a situation close to real prosthetic use (in which stimuli may appear with little intervals in between), and also to obtain unbiased results for classification (e.g., not to obtain the high classification just by recognition of signal versus rest).

Signals recorded from single-channel cuff electrodes could be used to discriminate sensory stimuli depending on their physiological nature. The pain fibers are of A*δ *(diameter 2-5 *μ*m) and C (0.3-1.3 *μ*m) types, and do not overlap in size and conduction velocity with fibers conveying the other stimuli tested. Cutaneous low-threshold mechanoreceptors (touch perception) use A*β *fibers (6-12 *μ*m) for conduction, whereas proprioceptive fibers correspond to A*α *(12-22 *μ*m) and A*β *types. Nerve fiber diameter is proportional to conduction velocity and signal strength, hence it would be expected that the best discrimination should be obtained among the stimuli that use different types of fibers for conduction, and that distinction between stimuli conducted by the same type of fibers would be difficult, due to the summing nature of cuff electrode recordings. However, in our classifications it was possible to distinguish proprioceptive and tactile stimuli, and in less cases nociceptive signals. Proprioceptive signals induced by fast toe flexion were likely transmitted by A*α *fibers from muscle spindles primary afferents, thus distinguishable from A*β *touch fibers. On the other hand, regarding the identification of the three stimuli, classification errors were mostly due to pain signals, which are conducted by thin nerve fibers, thus producing signals of small amplitude and difficult to visualize in the raw recordings.

In order to test our hypothesis that stimuli conducted by the same type of fibers are difficult to discriminate, two other types of touch stimuli were used during the experiments: light touch with a hair brush and fast scratch with a plastic probe. When performing the analysis between two and three types of tactile stimuli (results not shown), the performance became very poor, in accordance to our hypothesis that it is possible to distinguish different types of stimuli only if they correspond to different sensory modalities.

Our results show that it may be possible to develop robust, although limited, closed-loop control algorithms for neuroprostheses by means of the sensory information extracted with single-channel cuff electrodes from the peripheral nerve. Better results could be eventually achieved by using multi-polar epineural [38,39] and intraneural [40,41] electrodes, the latter being also more invasive. A limitation of our experimental procedure is that the experiments were performed in anesthetized animals, therefore we prevented problems regarding EMG signals due to animal movements and neural efferent signals, which, in real prostheses, would be mixed with afferent signals. While the EMG signal can be suppressed by digital filters (as in the present work) or by passive networks [42], the discrimination among efferent and afferent signals is more challenging, and should be tested in freely moving animals or in human implants.

An interesting aspect is that the analyses were performed on integral datasets, with training on first half and testing on second half of data, attempting to understand the real-life applicability of the single-channel cuffs.

Since an important feature for the correct classification of neural signals is a significant difference in terms of SNR for the different stimuli - this depending on implant position that is a relatively blind procedure - systems for navigation, which search for the best SNR achievable during implantation, may improve the results of this approach, similarly to the suggestions made for intraneural electrodes [43].

## Conclusions

This paper aimed at understanding the potential application of nerve signals recorded by means of single-channel cuff tripolar electrodes for identifying natural sensory information, in continuous-time applications, on integral datasets obtained from acute rat experiments. The signals from rat nerves were processed (obtaining optimal values for different signal processing parameters), and the results indicate that signals of acceptable SNR and corresponding to different physiological modalities (e.g. mediated by different types of nerve fibers) may be distinguished. By means of power-based features and an artificial classifier, proprioceptive and touch signals conducted by different fiber types were distinguished; instead, although conducted by other fibers, pain signals, due to their low SNR, were difficult to discriminate consistently.

## Competing interests

The authors declare that they have no competing interests.

## Authors' contributions

SM and XN designed the experimental protocol and supervised all the scientific activities. SR and JC developed the processing and pattern recognition algorithms. SR, EU and XN performed the animal in-vivo experiments. All authors wrote, read, and approved the final manuscript.
